# Efficacy and safety of rituximab treatment in early primary Sjögren's syndrome: a prospective, multi-center, follow-up study

**DOI:** 10.1186/ar4359

**Published:** 2013-10-30

**Authors:** Francesco Carubbi, Paola Cipriani, Alessandra Marrelli, Paola Di Benedetto, Piero Ruscitti, Onorina Berardicurti, Ilenia Pantano, Vasiliki Liakouli, Saverio Alvaro, Alessia Alunno, Antonio Manzo, Francesco Ciccia, Roberto Gerli, Giovanni Triolo, Roberto Giacomelli

**Affiliations:** 1 Department of Clinical Science and Biotechnology, Rheumatology Unit, University of L’Aquila, 67100 L’Aquila, Italy; 2 Department of Clinical and Experimental Medicine, Rheumatology Unit, University of Perugia, 06122 Perugia, Italy; 3 Division and Laboratory of Rheumatology, University of Pavia, 27100 Pavia, Italy; 4 Department of Internal Medicine, Rheumatology Unit, University of Palermo, 90127 Palermo, Italy

## Abstract

**Introduction:**

Primary Sjögren’s syndrome (pSS) is an autoimmune disorder affecting exocrine glands; however, a subgroup of pSS patients experience systemic extra-glandular involvement leading to a worsening of disease prognosis. Current therapeutic options are mainly empiric and often translated by other autoimmune diseases. In the last few years growing evidence suggests that B-cell depletion by rituximab (RTX) is effective also in pSS. Patients with early active disease appear to be those who could benefit the most from RTX. The aim of this study was to investigate the efficacy and safety of RTX in comparison to disease modifying anti-rheumatic drugs (DMARDs) in early active pSS patients.

**Methods:**

Forty-one patients with early pSS and active disease (EULAR Sjogren’s syndrome disease activity index, ESSDAI ≥ 6) were enrolled in the study. Patients were treated with either RTX or DMARDs in two different Rheumatology centers and followed up for 120 weeks. Clinical assessment was performed by ESSDAI every 12 weeks up to week 120 and by self-reported global disease activity pain, sicca symptoms and fatigue on visual analogic scales, unstimulated saliva flow and Schirmer’s I test at week 12, 24, 48, 72, 96, and 120. Laboratory assessment was performed every 12 weeks to week 120. Two labial minor salivary gland (MSG) biopsies were obtained from all patients at the time of inclusion in the study and at week 120.

**Results:**

Our study demonstrated that RTX treatment results in a faster and more pronounced decrease of ESSDAI and other clinical parameters compared to DMARDs treatment. No adverse events were reported in the two groups. We also observed that RTX is able to reduce glandular infiltrate, interfere with B/T compartmentalization and consequently with the formation of ectopic lymphoid structures and germinal center-like structures in pSS-MSGs.

**Conclusions:**

To our knowledge, this is the first study performed in a large cohort of early active pSS patients for a period of 120 weeks. We showed that RTX is a safe and effective agent to be employed in pSS patients with systemic, extra-glandular involvement. Furthermore, our data on pSS-MSGs provide additional biological basis to employ RTX in this disease.

## Introduction

Primary Sjögren’s syndrome (pSS) is a systemic autoimmune disease characterized by mucosal dryness in the majority of patients. General symptoms of pSS include fatigue, weight loss and fever [1,2], and extraglandular manifestations occur in 20 to 40% of patients affecting the musculoskeletal system, skin, peripheral and central nervous system, kidneys, lungs [3] and health-related quality of life (HR-QoL) [4].

Classically, minor salivary glands (MSGs) of patients with pSS show a focal lymphocytic sialadenitis (FLS) characterized by the presence of one or more dense aggregates of 50 or more lymphocytes usually located in perivascular or periductal areas. The infiltrating lymphocytes are often organized into tertiary lymphoid tissues in nonlymphoid locations, also known as ectopic lymphoid structures showing a network including specific segregated T-cell and B-cell zones and follicular dendritic cells. Some of these tertiary lymphoid tissues are arranged in germinal centers (GC) [5,6].

Concerning pSS pathogenesis, B-lymphocyte hyperactivity, MSG infiltration and development of B-cell follicles containing GC-like structures represent the hallmarks of the disease. Although the peculiar role of B cells in autoimmunity is the production of autoantibodies, growing evidence suggest that B cells may also exert additional pivotal functions such as antigen presentation and release of specific cytokines with immune regulatory, proinflammatory, polarizing and tissue-organizing functions [7,8].

Prolonged B-cell survival and aberrant B-cell activity may lead to the development of non-Hodgkin lymphomas in 5% of pSS patients, and among these extranodal marginal zone B-cell lymphoma of the mucosa-associated lymphoid tissue represents the most common subtype [9]. Strong predictors of lymphoproliferative disorder in pSS include parotid, lymph node and/or splenic enlargement, monoclonal gammopathy, hypogammaglobulinemia, mixed cryoglobulinemia, palpable purpura, CD4^+^ T-cell lymphopenia and/or reduced levels of C4 [10].

B-cell activating factor (BAFF), also named B-lymphocyte stimulator, is an important member of the tumor necrosis factor family and is involved in B-cell survival and humoral immune responses, playing a critical role in B-cell homeostasis [11]. BAFF overexpression rescues autoreactive cells from depletion in periphery, leading to a higher number of mature autoreactive B cells [12]. Patients with pSS display elevated levels of BAFF in serum, saliva and salivary glands [13-15]. In their sera, BAFF levels correlate with gamma-globulin levels, anti-SSA and/or anti-SSB antibodies. Furthermore, higher BAFF levels are observed in patients with GCs in salivary glands [16,17]. Evidence from animal models and patients with pSS demonstrated that the formation and maintenance of tertiary lymphoid tissues is critically dependent on ectopic expression of lymphotoxins and homeostatic chemokines such as CXCL13 and CXCL12 and interaction with their specific receptors, CXCR5 and CXCR4 respectively. These key molecules are produced during chronic inflammation by several cell types, including resident epithelial, stromal and endothelial cells, as well as different subsets of infiltrating immune cells [18]. Moreover, such lymphoid chemokines exert a critical role during lymphoid organogenesis, allow the transformation of diffuse infiltrates into highly organized structures that are necessary for the initiation of the adaptive immune response, and, as already mentioned, play an important role in tertiary lymphoid tissue development [19].

The lymphotoxin-alpha (LTα) and lymphotoxin-beta (LTβ) pathways have been associated with the presence of ectopic lymphoid structures at the sites of chronic inflammation in several autoimmune diseases [20]. Furthermore, when compared with the inflammatory process of nonspecific sialoadenitis, salivary glands of patients with pSS have been found to express a unique profile of adhesion molecules, cytokines and chemokines, including a striking overexpression of CXCL13 and, to a lesser degree, CXCL12 [5,21-23]. B cells expressing the corresponding receptors for CXCL13 and CXCL12 have been detected in the peripheral blood and glandular infiltrates of pSS patients [24,25]. The striking glandular overexpression of CXCL12 and CXCL13 in pSS patients might contribute to the preferential migration of CXCR4 and CXCR5 expressing CD27^+^ memory B cells into the inflamed salivary glands, resulting in lower numbers of circulating CD27^+^ memory cells [24]. Furthermore, CXCL12–CXCR4 and CXCL13–CXCR5 interactions have been widely suggested to be involved in B-cell disturbance during pSS and may be closely associated with the whole inflammatory process, the development of ectopic GC-like structures as well as the peripheral B-cell abnormalities [25].

Treatment of pSS is largely empiric and in the last 50 years has been mainly symptomatic. In this setting, systematic reviews highlighted the limited evidence available for the drugs that are frequently used in pSS and the difficulties in elaborating solid therapeutic recommendations [26]. The increasing availability of targeted therapies in the last two decades, mirroring what happened in rheumatoid arthritis, raised the possibility in pSS to selectively interfere with different pathogenic pathways. On this basis, B-cell depletion by rituximab (RTX) represents an issue of great interest. RTX is a chimeric humanized monoclonal anti-CD20 antibody. CD20 is expressed on the surface of pre-B lymphocytes, transitional B lymphocytes and mature B lymphocytes, and is lost at the plasma cell stage [27]. CD20 mediates B-cell activation, proliferation and differentiation [28] and plays an important role in the generation of T-cell-independent antibody responses [29].

Although few data are present in literature to date, evidence from both open-label studies and randomized clinical trials provide the clue that RTX is effective in reducing various disease manifestations of pSS patients, such as sicca symptoms, extraglandular manifestations and fatigue, and in improving saliva production and, where explored, HR-QoL [28,30,31]. Following B-cell depletion therapy by RTX, an increase in serum levels of BAFF was observed in pSS; and to note, higher baseline serum levels of BAFF resulted in a shorter duration of B-cell depletion by RTX, indicating a role of BAFF in the repopulation of B cells after RTX treatment [32,33].

Regarding the effect of RTX therapy on the levels of LTα, LTβ and the chemokine–chemokine receptor pairs CXCL13–CXCR5 and CXCL12–CXCR4, in patients affected by pSS, no data are currently available in the literature. Studies reporting the effects of RTX treatment in rheumatoid arthritis, multiple sclerosis and B-cell malignancies allow one to speculate that similar effects could occur during pSS. In particular, serum levels of CXCL13 were found to be predictive for the repopulation, following a single course of RTX in rheumatoid arthritis. A direct correlation between serum and synovial CXCL13 was also observed in these patients [34].

Although B-cell-depleting therapy with RTX provided promising results, the underlying mechanisms of action are not completely understood and, it is still unclear why some pSS patients fail to achieve a good clinical response. In this setting, one must point out that patients with residual salivary function and treated in early phases of the disease showed a better response concerning eye dryness, fatigue and HR-QoL [35,36]. Consistent clinical improvement was also reported for both glandular enlargement [37] and extraglandular manifestations [31,36,38-40]. On this basis, we can speculate that pSS patients with early, active disease, experiencing extraglandular manifestations, might strongly benefit from RTX treatment.

The aim of this prospective, multi-center, follow-up study was to assess the safety and efficacy of RTX, compared with treatment with disease-modifying anti-rheumatic drugs (DMARDs), in recent-onset pSS patients, with active disease, by a follow-up period of 120 weeks. MSG biopsy was employed in the study both to confirm the diagnosis of pSS and to evaluate histological and molecular modification in the tissues, following treatment. Clinical response was evaluated by validated measures.

## Methods

### Study population

Forty-one consecutive patients referring to the Rheumatology Unit, University of L’Aquila, Italy, or to the Rheumatology Unit, University of Palermo, Italy, were enrolled. All patients were over 18 years old and fulfilled the American–European consensus criteria for pSS, including histopathologic criteria [41]. Inclusion criteria were: pSS diagnosis, recent onset of the disease (maximum 2 years from the onset of first symptoms related to the disease) and active disease, as defined by values >50 mm for two out four visual analogical scales (VAS; 0 to 100 mm) such as: global disease activity (including extraglandular manifestations); pain; sicca symptoms; and fatigue, and a EULAR Sjögren’s syndrome disease activity index (ESSDAI) [42] arbitrarily chosen ≥6. Patients were excluded for secondary Sjögren’s syndrome, severe cardiac, pulmonary, renal or hematologic failure, a history of cancer in the last 5 years, hepatitis B or hepatitis C infection, human immunodeficiency virus infection, tuberculosis, severe diabetes, and any other chronic disease, or evidence of infection, and if they were unable to understand and to adhere to the protocol. Treatment with DMARDs and corticosteroids was discontinued at least 6 months before baseline, except for these patients with severe extraglandular manifestations needing continuation of treatment (with no change in dosage allowed). The use of any systemic drug for the relief of dryness-related symptoms was not allowed during the study.

### Study design and sample size

This is a prospective, multi-center, follow-up study. The whole study was approved by the local ethics committee of the study coordinator (Local Health Authority ASL 1 Avezzano-Sulmona-L’Aquila Ethic Committee) and performed according to the Good Clinical Practice guidelines. All patients provided written informed consent in accordance with the declaration of Helsinki. Patients enrolled by the Rheumatology Unit of L’Aquila received RTX therapy with a stable dose of prednisone (RTX arm, 19 patients) and patients enrolled by the Rheumatology Unit of Palermo were treated with one or more DMARDs (hydroxychloroquine, methotrexate, cyclosporine) that are largely used in this disease, although no one of these has been licensed for pSS, in association with a stable dose of prednisone (DMARD arm, 22 patients), according to clinical manifestations. Based on a formal sample size calculation, 41 patients were included, of whom 19 were assigned to receive RTX and 22 to receive DMARD treatment. The duration of the study was 120 weeks.

### Rituximab administration

Patients in the RTX arm received infusion of 1,000 mg rituximab (Mabthera®; F. Hoffmann-La Roche AG, Basel, Switzerland) at day 1 and at day 15 to complete a course of therapy. This course was repeated every 24 weeks. During the study period, all patients received six courses of therapy with RTX. To minimize side effects, all patients were pretreated with methylprednisolone (40 mg intravenously), paracetamol (1,000 mg orally) and chlorpheniramine (10 mg intravenously).

### Study endpoints

The primary endpoints of this study were: the evaluation of the safety of RTX infusion; and a significant variation in the reduction of the ESSDAI in the RTX arm during the study period. Secondary endpoints were measurements of salivary/lacrimal function, subjective variables and biologic effects of RTX in MSGs, compared with patients treated with DMARD therapy.

### Clinical assessment

The disease activity was assessed using the ESSDAI at baseline and every 12 weeks to week 120. The following self-reported outcomes on VAS (0 to 100 mm), evaluated at baseline and weeks 12, 24, 48, 72, 96, and 120, were employed: global disease activity (including extraglandular manifestations), pain, sicca symptoms and fatigue. An independent blind clinician provided the degree of physician global assessment of the disease activity by VAS, evaluated at baseline and weeks 12, 24, 48, 72, 96, and 120. Unstimulated saliva flow and Schirmer’s I test for ocular function were performed at baseline and weeks 12, 24, 48, 72, 96, and 120, as described previously [43,44]. The IgG concentration, antinuclear antibodies, anti-Ro/SSA and anti-La/SSB antibodies, and rheumatoid factor were also assessed at baseline and weeks 12, 24, 48, 72, 96, and 120.

### Histological analysis of minor salivary gland biopsies

Two labial MSG biopsies were obtained from all patients at the time of inclusion in the study and at week 120. Five healthy donors (four women and one man) with sicca symptoms but normal MSGs and absence of any clinical and serological feature of Sjögren’s syndrome acted as controls. These samples were divided into two parts: the first for histologic analysis, and the second for RNA isolation and quantitative real-time polymerase chain reaction. For histologic evaluation, hematoxylin–eosin-stained sections were examined by a blinded, trained operator. We performed both the Chisholm and Mason score (grades 1 to 4) [45] and the focus score, the latter assessing the number of foci/4 mm^2^ (a focus is defined as an aggregate >50 mononuclear cells) [46]. The cellular infiltrate and the lymphoid organization were assessed by immunofluorescence staining of sequential sections with monoclonal antibodies recognizing CD3, CD20, CD21 (all provided by Dako Cytomation, Glostrup, Denmark), CXCR4, CXCL12, CXCR5, and CXCR13 (all provided by R&D System, Abingdon, UK). Formalin-fixed, paraffin-embedded sections measuring 3 μm in thickness were dewaxed and rehydrated. All sections underwent high-temperature antigen retrieval using Target Retrieval Solution (Dako, Glostrup, Denmark) for 35 minutes at 95°C, except for sections stained with anti-CD21 that underwent proteolytic digestion for 7 minutes at 37°C using proteinase K (Dako).

For sections stained with anti-CD3, anti-CD21, anti-CXCR5 or anti-CXCL12 antibodies, we performed nonspecific binding blocking (protein block serum-free ready-to-use; Dako). For sections stained with anti-CD20, anti-CXCR4 or CXCL13 antibodies, we also performed endogenous biotin blocking (avidin–biotin blocking system; Vector Laboratories, Burlingame, CA, USA).

To assess T-cell/B-cell segregation, sections were double-stained with anti-CD3/anti-CD20 antibodies. Monoclonal mouse anti-human CD3 (dilution 1:50) was incubated for 1 hour at room temperature in a wet chamber, followed by 30 minutes incubation with secondary antibody goat anti-mouse/AlexaFluor-555 (1:100; Invitrogen, Paisley, UK). Subsequently, monoclonal mouse anti-human CD20 (1:20) incubation was performed for 1 hour at room temperature in a wet chamber, followed by 30 minutes incubation with biotin goat anti-mouse IgG (1:100; Biolegend, San Diego, CA, USA) and 30 minutes incubation with streptavidin/AlexaFluor-488 (1:200; Invitrogen).

To detect follicular dendritic cell networks and identify GCs, sections were incubated overnight at 4°C with monoclonal mouse anti-human CD21 (1:20) followed by 30 minutes incubation with secondary antibody goat anti-mouse/AlexaFluor-488 (1:100; Invitrogen).

To assess the presence of CXCR4 in MSGs, sections were stained with anti-CXCR4 antibody. Monoclonal mouse anti-human CXCR4 (dilution 1:50) was incubated for 1 hour at room temperature in a wet chamber, followed by 30 minutes incubation with biotin antibody goat anti-mouse IgG (1:100; Biolegend) and 30 minutes incubation with streptavidin/AlexaFluor-488 (1:200; Invitrogen).

CXCL12 positivity was evaluated by a specific anti-CXCL12 antibody. Monoclonal mouse anti-human CXCL12 (1:20) incubation was performed overnight at 4°C in a wet chamber, followed by 30 minutes incubation with secondary antibody goat anti-mouse/AlexaFluor-555 (1:100; Invitrogen).

Anti-CXCR5 antibody was employed to assess the evidence of CXCR5 in the studied samples, which were incubated with monoclonal mouse anti-human CXCR5 (dilution 1:50) overnight at 4°C in a wet chamber, followed by 30 minutes incubation with secondary antibody goat anti-mouse/AlexaFluor-555 (1:100; Invitrogen).

Anti-CXCL13 antibody was employed to assess the evidence of CXCL13 in the studied samples, which were incubated with monoclonal mouse anti-human CXCL13 (dilution 1:50) for 1 hour at room temperature in a wet chamber, followed by 30 minutes incubation with biotin antibody goat anti-mouse IgG (1:100; Biolegend) and 30 minutes incubation with streptavidin/AlexaFluor-555 (1:200; Invitrogen).

All sections were counterstained with 4′, 6-diamidino-2-phenylindole (1:1,000) for 10 minutes and slides were mounted with Mowiol 4–88 (Sigma-Aldrich, Milan, Italy). Images were acquired using an Olympus BX53 fluorescence microscope with CellSens software (Olympus America Inc., Center Valley, PA, USA). The number of CXCR4-positive and CXCR5-positive cells was determined as follows: three random high-power microscopic fields for each area, showing an immune-inflammatory infiltrate, were selected and the numbers of CXCR4 and CXCR5 immunoreactive cells were counted at 200× magnification. The mean values of positive cells in each area were added and divided for the number of areas in the section [47].

### RNA isolation and quantitative real-time polymerase chain reaction

Total RNA was extracted from MSGs using TRIZOL (SIGMA, St. Louis, MO, USA) and reverse transcribed into complementary DNA (cDNA) with the ThermoScript reverse transcription–polymerase chain reaction system (Invitrogen, Carlsbad, CA, USA). The quantitative real-time polymerase chain reaction was performed using SYBR green kits and Taqman gene expression assays (Applied Biosystems, Utrecht, the Netherlands). Results were analyzed after 40 cycles of amplification using the ABI 7500 Fast Real Time PCR System (Applied Biosystems, Utrecht, the Netherlands). Primers were designed on the basis of the reported sequences (Primer bank NCBI): LTα, 5′-ATGACACCACCTGAACGTCTC-3′ (forward) and 5′-CTCTCCAGAGCAGTGAGTTCT-3′ (reverse); LTβ, 5′-GACGAAGGAACAGGCGTTTCT-3′ (forward) and 5′-GTAGCCGACGAGACAGTAGAG-3′ (reverse); β-actin, 5′-CCTGGCACCCAGCACAAT-3′ (forward) and 5′-AGTACTCCGTGTGGATCGGC-3′ (reverse); CXCR5, 5′-CACGTTGCACCTTCTCCCAA-3′ (forward) and 5′-CGCCACATGGTAGAGGAATCG-3′ (reverse); CXCL13, 5′-GCTTGAGGTGTAGATGTGTCC-3′ (forward) and 5′-CGATCAATGAAGCGTCTAGGGAT-3′ (reverse); CXCR4, 5′-GACTTGTGGGTGGTTGTG-3′ (forward) and 5′-AGGATGAGGATGACTGTGG-3′ (reverse); CXCL12, 5′-GAGCCAACGTCAAGCATCTC-3′ (forward) and 5′-CAATGCACACTTGTCTGTTG-3′ (reverse); and BAFF, 5′-TTGAGTCTGGTGACTTTGTTTCG-3′ (forward) and 5′-GCAAGTTGGAGTTCATCTCCTT-3′ (reverse).

### Statistical analysis

SPSS 13.0 software (IBM, Armonk, NY, USA) was used for statistical analysis. One-way analysis of variance and multiple comparison *post-hoc* tests were employed to calculate differences between baseline and the following time points. Differences between the two treatment arms were tested with nonparametric Mann–Whitney U test. The Wilcoxon matched-pairs test was used to compare variables at baseline and week 120. When required, the chi-square test was also employed. *P* <0.05 was considered significant.

## Results

### Baseline demographic and clinical characteristics

Demographic and clinical characteristics of the patients at baseline were balanced across the RTX and DMARD groups (Table 1). The mean age of the patients in DMARD group was 43 years and in the RTX group was 40 years, while the mean disease duration was 14 and 13 months in the DMARD and RTX groups, respectively. The mean ESSDAI score at baseline was 19.8 in the DMARD group and 20.3 in the RTX group, with an equal proportion of patients with similar clinical features in each group.

**Table 1:** Demographic, clinical and histological characteristics of the patient cohort

**Treatment group**		
	**DMARDs**	**RTX**
Number of patients	22	19
Age (years)	43 (30 to 56)	40 (27 to 53)
Sex (female)	22 (100%)	18 (94.7%)
Disease duration (months)	14 (7 to 21)	13 (6 to 20)
Parotid gland enlargement	18 (81.8%)	16 (84.2%)
Cutaneous vasculitis	2 (9.0%)	3 (15.7%)
Pulmonary involvement	1 (4.5%)	1 (5.2%)
Neurological involvement	3 (13.6%)	2 (10.5%)
Renal involvement	0 (0%)	0 (0%)
Autoimmune cytopenia	7 (31.8%)	8 (42.1%)
Myositis	0 (0%)	0 (0%)
Arthritis.	11 (50%)	9 (47.3%)
Lymphadenopathy	15 (68.1%)	17 (89.4%)
ESSDAI	19.8 (6 to 41)	20.3 (6 to 41)
Prednisone^a^	22 (12.5)	19 (12.5)
DMARDs (HCQ/MTX/CsA)	22 (15/13/8)	0 (0/0/0)
Chisholm and Mason score ≥3	22	19
Focus score	1.8 (1 to 3.3)	1.8 (1 to 3.4)

Data presented as mean (range) or number (%) unless otherwise stated. CsA, cyclosporine; DMARDs, disease-modifying anti-rheumatic drugs; HCQ, hydroxychloroquine; MTX, methotrexate; RTX, rituximab. ^a^Data presented as number of patients (mean dose in milligrams).

### Primary endpoints

**Figure 1:**
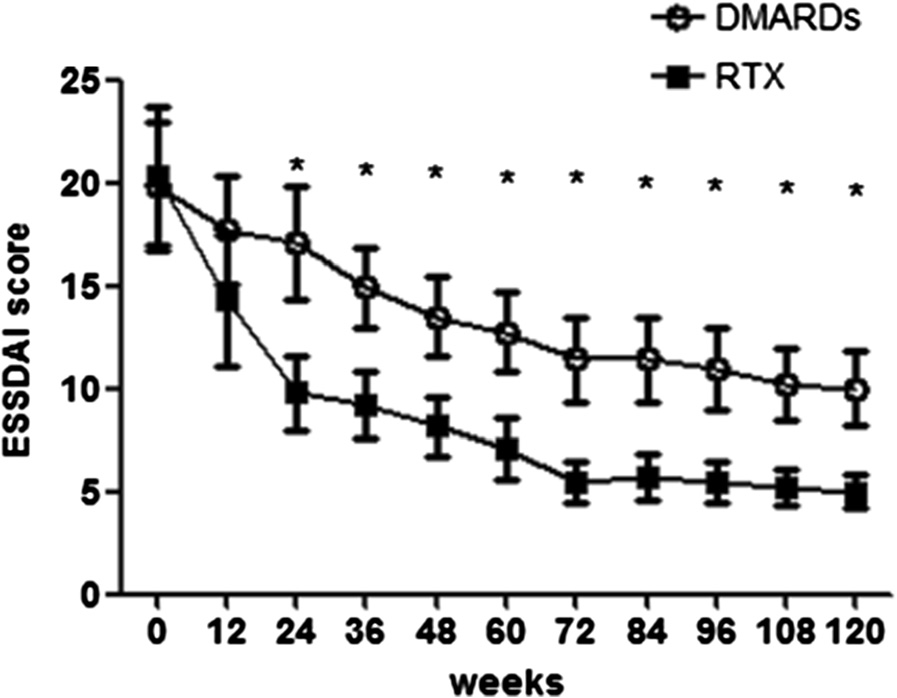
**Primary endpoint of the study EULAR Sjögren’s syndrome disease activity index in the rituximab and disease-modifying anti-rheumatic drug treatment groups.** Primary endpoint of the study EULAR Sjögren’s syndrome disease activity index (ESSDAI) in the rituximab (RTX) and the disease-modifying anti-rheumatic drug (DMARD) treatment groups. Bars in the line charts indicate mean ± standard error of the mean. *P < 0.05, comparison between the two treatment arms at the corresponding time point.

Our study clearly shows that RTX treatment, already from the second course of therapy, displayed a stronger and significant effect in decreasing the ESSDAI, when compared with the DMARD arm, and this effect was observed throughout the study period (Figure 1 and Table 2). Mean change of ESSDAI from baseline to week 24 was significantly greater with RTX than with DMARD therapy (Table 2). Mean values for ESSDAI were similar at baseline and decreased sharply by week 12 in both groups. Thereafter, the ESSDAI response curves separated from week 24 (Figure 1).

**Table 2:** Clinical assessment result changes from baseline to week 120 in the rituximab and DMARD treatment groups

	**BASELINE**		**Week 12**		**Week 24**		**Week 48**		**Week 72**		**Week 96**		**Week 120**	
	**DMARDs**	**RTX**	**DMARDs**	**RTX**	**DMARDs**	**RTX**	**DMARDs**	**RTX**	**DMARDs**	**RTX**	**DMARDs**	**RTX**	**DMARDs**	**RTX**
**Global disease activity VAS (mm)**	88.7 ± 3.3	88.7 ± 3.3	77 ± 2.2†	72.4 ± 4.0‡	71 ± 3.2‡	68.5 ± 4.6‡	63 ± 3.9*	60.0 ± 4.6*	54 ± 3.5*	51.8 ± 3.0*	50.5 ± 3.7*	47.1 ± 3.5*	52.1 ± 4.8*	45.3 ± 3.9*
**Pain VAS (mm)**	70.1 ± 9.0	71.1 ± 8.7	59.8 ± 7.8†	55 ± 7.4†	56.7 ± 7.8‡	53.5 ± 7.5‡	51.3 ± 7.5*	44.1 ± 8.5*	41 ± 7.6*	36.3 ± 7.9*	40.5 ± 7.5*	33.1 ± 7.0*	39.3 ± 8.3*	29.8 ± 7.8*
**Fatigue VAS (mm)**	85.1 ± 4.6	83.8 ± 4.1	73 ± 4.5†	63.1 ± 4.3‡	63.1 ± 4.7‡	58.7 ± 5.1‡	60.1 ± 4.4*	56.2 ± 5.5*	52.2 ± 4.0*	47.8 ± 4.7*	50.5 ± 3.8*	42.5 ± 4.1*	51.8 ± 4.5*	41.1 ± 4.2*
**Dryness VAS (mm)**	71.0 ± 8.3	72 ± 8.5	61.1 ± 8.3	55.3 ± 7.6‡	58 ± 8.2†	52.2 ± 7.6‡	56.3 ± 8.4‡	44.1 ± 8.5*	53.8 ± 11.0*	36.3 ± 7.9*	51.8 ± 10.4*	30.7 ± 8.0*	51.8 ± 11.1*	25.1 ± 7.7*
**Physician GA VAS (mm)**	89.7 ± 3.0	87.1 ± 2.3	78.6 ± 2.6†	69.1 ± 3.0†	73.1 ± 3.4‡	65 ± 4.4‡	65.0 ± 3.9*	56.6 ± 4.3*	56.6 ± 3.8*	50.3 ± 3.0*	54.7 ± 4.5*	45.8 ± 3.3*	56.1 ± 4.9*	44.0 ± 3.7*
**Unstimulated salivary flow (ml/minute)**	0.08 ± 0.03	0.08 ± 0.04	0.1 ± 0.04	0.2 ± 0.04‡	0.1 ± 0.04	0.3 ± 0.04‡	0.1 ± 0.07	0.4 ± 0.02*	0.1 ± 0.06	0.3 ± 0.001*	0.1 ± 0.08	0.4 ± 0.04*	0.1 ± 0.08	0.4 ± 0.04*
**Schirmer I test (mm/5 minutes)**	3.8 ± 0.6	3.8 ± 0.6	4 ± 0.5	5.5 ± 0.4‡	4.5 ± 0.7	6.0 ± 0.5†	5.1 ± 0.8	6.3 ± 0.6‡	5.1 ± 0.7	7.0 ± 0.7*	5.3 ± 0.7†	7.0 ± 0.7*	5.5 ± 0.8‡	7.3 ± 0.8*
**ESSDAI**	19.8 ± 3.1	20.3 ± 2.9	17.6 ± 3.2	14.2a ± 2.8	14.2 ± 3.0‡	9.8 ± 2.0‡	12.3 ± 2.0*	9.4 ± 1.6‡	10.2 ± 2.0*	5.7 ± 1.0*	9.7 ± 2.0*	5.7 ± 1.0*	8.8 ± 1.7*	5.2 ± 0.9*

Data presented as mean ± standard error of the mean. DMARD, disease-modifying anti-rheumatic drug; ESSDAI, EULAR Sjögren’s syndrome disease activity index; GA, global assessment; RTX, rituximab; VAS, visual analogic scale. *P <0.001;‡*P* <0.01; †*P* <0.05.

In both treatment groups, no adverse events were reported during the study. The RTX infusions were generally well tolerated in this study and no local or systemic infusion reactions were observed. No adverse events judged by the investigator to be possibly, probably, or definitely related to RTX therapy were observed in the 120-week follow-up period. No patient needed dose modification or interruption because of adverse events, and no patients withdrew from the study.

### Clinical assessment

**Figure 2:**
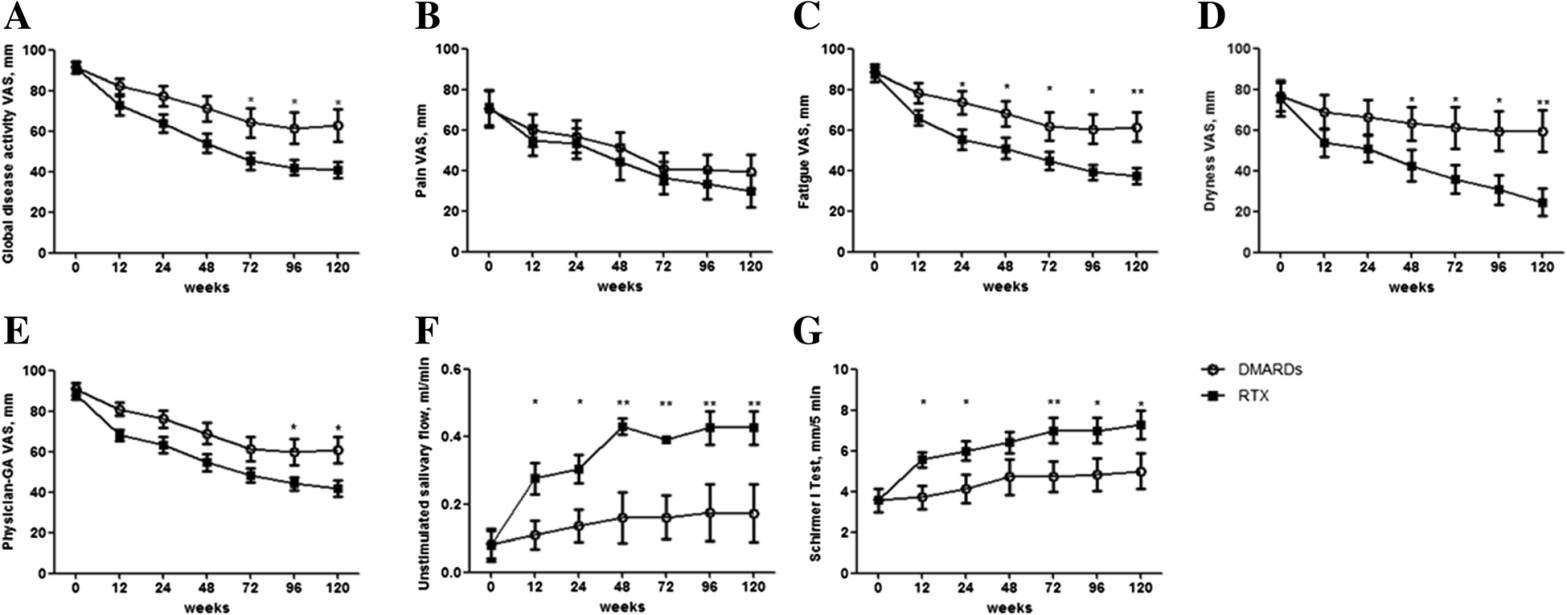
**Clinical assessment in the rituximab and disease-modifying anti-rheumatic drug treatment groups.** Clinical evaluation was performed as follows: **(A)** global disease activity visual analogic scale (VAS), **(B)** pain VAS, **(C)** fatigue VAS, **(D)** dryness VAS, **(E)** physician global assessment (GA) VAS, **(F)** unstimulated salivary flow, and **(G)**Schirmer I test. Bars in the line charts indicate mean ± standard error of the mean. **P <0.01 and *P <0.05, comparison between the two treatment arms at the corresponding time point. DMARD, disease-modifying anti-rheumatic drug; RTX, rituximab.

The response curves for VAS global disease activity, VAS pain and physician global assessment showed much the same pattern of ESSDAI (Figure 2A,B,E and Table 2). In fact, these items significantly decreased in both groups, and again the RTX arm showed a better performance when compared with the DMARD population. Such decrease was progressive up to week 72 and then reached a plateau until week 120.

As far as VAS dryness was concerned, we observed in the RTX treatment group a progressive improvement of its score from baseline to week 120 (Figure 2D). On the contrary, only a slight decrease in VAS dryness was observed in the DMARD treatment group until week 12, but no further amelioration were detected thereafter. The results concerning VAS fatigue mirrored those observed for VAS dryness (Figure 2C). As far as the objective measurements of salivary and lacrimal gland function were concerned, we observed that both the unstimulated salivary flow and the Schirmer’s I test were not affected in the DMARD treatment group (Figure 2F,G and Table 2). On the contrary, RTX was able to significantly increase both tests from week 12. Concerning serological parameters, we did not observe any significant difference in IgG concentration, antinuclear antibodies, rheumatoid factor and anti-Ro/SSA and anti-La/SSB antibodies in each arm at any time point and between the two studied groups, before and after treatment (data not shown).

### Histological analysis of minor salivary gland biopsies

**Figure 3:**
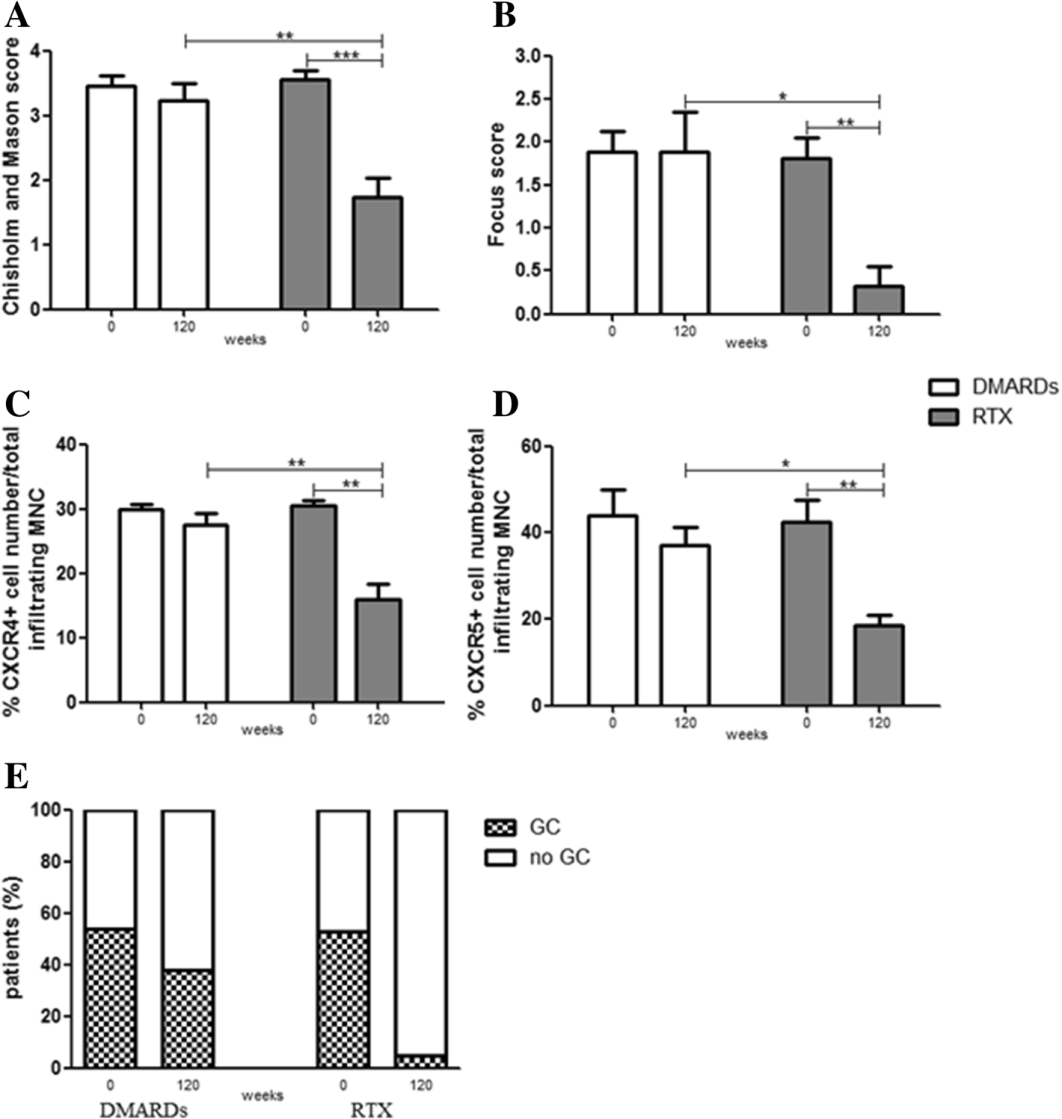
**Histological assessment of minor salivary gland biopsies. (A)**, **(B)** Histological scoring of minor salivary gland (MSG) biopsies in the rituximab (RTX) and disease-modifying anti-rheumatic drug (DMARD) treatment groups at baseline and week 120. **(C)**, **(D)** Percentage of CXCR4-positive cells and CXCR5-positive cells inside the glandular infiltrate. Bars indicate mean ± standard error of the mean. ***P <0.001, **P <0.01 and *P <0.05. **(E)** Percentage of patients displaying germinal centers (GCs) in the RTX and DMARD treatment groups at baseline and week 120. MNC, mononuclear cells.

**Figure 4:**
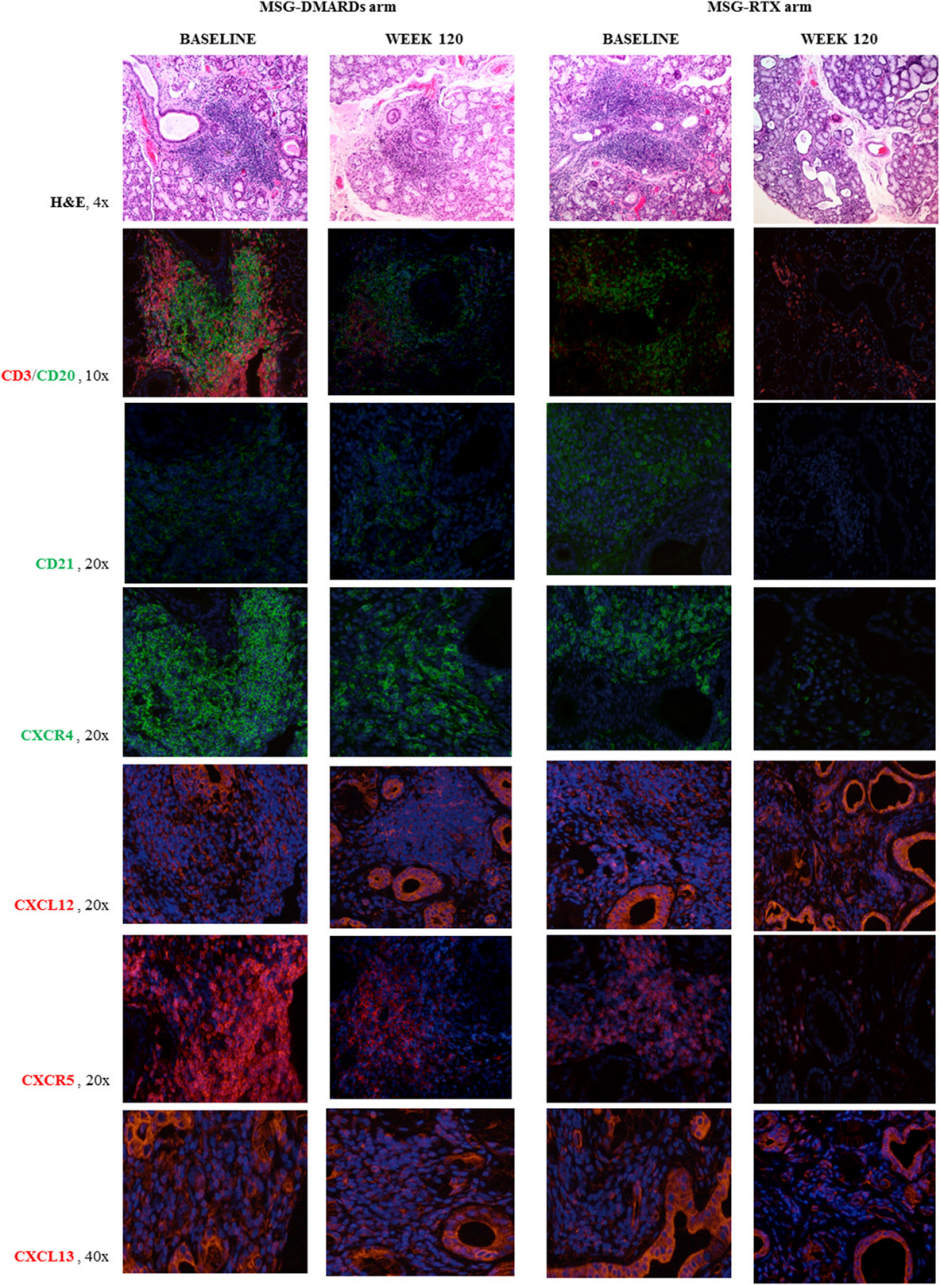
**Histological analysis of minor salivary gland biopsies in the rituximab and disease-modifying anti-rheumatic drug treatment groups.** Histological analysis of minor salivary gland (MSG) biopsies in the rituximab (RTX) and disease-modifying anti-rheumatic drug (DMARD) treatment groups at baseline and week 120. 4×, 10×, 20× and 40×, level of magnification of the corresponding picture H&E, hematoxylin–eosin.

At baseline, no significant difference in terms of focus score and Chisholm and Mason grading were observed between RTX-treated and DMARD-treated patients (Table 1). Twelve out of 22 patients in the DMARD group (54.5%) and 10 out of 19 patients in the RTX group (52.6%) displayed GC-like structures at baseline. As summarized in Figure 3A,B, both Chisholm and Mason grading and focus score were not modified at week 120 in patients treated with DMARDs while a significant reduction compared with baseline was observed in RTX-treated patients. In particular, in 17 out of 19 RTX-treated patients (89.4%) the foci number at week 120 was below 1, suggesting that RTX treatment might reverse the FLS, which is generally considered specific for pSS, to a nonspecific chronic sialadenitis pattern or, alternatively, leading to a full restoration of MSG architecture (Figure 4). Furthermore, we observed that the number of GC-positive biopsies dropped from 52.6% to 5.2% in RTX-treated patients, and after 30 months only one out of 19 patients still displayed GCs. On the contrary, no significance difference were observed in the DMARD group, where the number of GC-positive biopsies after treatment was slightly reduced (38.5%) and conversion from a FLS to a nonspecific chronic sialadenitis pattern was rarely observed (Figure 3E).

We also observed a reduction of CXCR4-positive and CXCR5-positive cell percentage in total infiltrating mononuclear cells when compared with baseline in RTX-treated patients but not in those treated with DMARDs at week 120 (Figure 3C,D). Figures 3 and 4 display a comparison between the two treatment arms for the aforementioned variables.

### Molecular analysis of minor salivary gland biopsies

**Figure 5:**
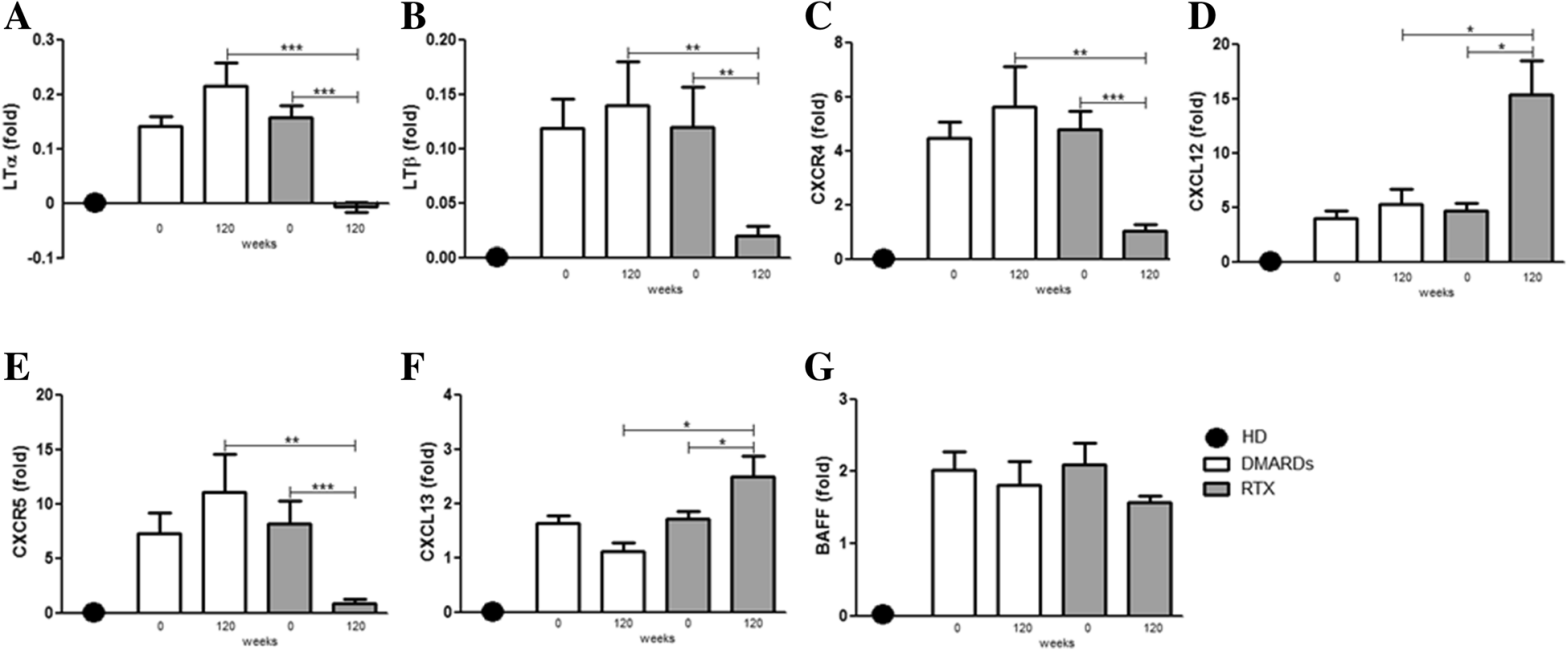
**Molecular analysis of minor salivary gland biopsies in the rituximab and disease-modifying anti-rheumatic drug treatment groups.** Relative mRNA quantification of **(A)** LTα, **(B)** LTβ, **(C)** CXCR4, **(D)** CXCL12, **(E)** CXCR5, **(F)** CXCL13, and **(G)** B-cell activating factor (BAFF) was assessed by quantitative real-time polymerase chain reaction in salivary glands obtained from 41 pSS patients and five healthy donors (HD). Bars indicate mean ± standard error of the mean. Levels are expressed as the fold increase or decrease in pSS MSGs compared with HD MSGs. ***P <0.001; **P <0.01 and *P <0.05. DMARD, disease-modifying anti-rheumatic drug; LT, lymphotoxin; RTX, rituximab.

To further support histological findings, we performed molecular analysis on the same biopsy specimens at baseline and week 120. Five normal MSGs of subjects with sicca symptoms and an absence of any clinical and serological feature of pSS were used as controls. At baseline, all of the patients, independent of treatment arm, displayed the same cytokine and chemokine patterns, which significantly differed from those observed in the MSGs of the subjects affected by sicca symptoms but without any feature of immune activation (Figure 5). As shown in Figure 5A,B, RTX treatment led to a significant reduction of LTα and LTβ expression and these results were not observed in patients treated with DMARDs. RTX treatment was also able to interfere with the CXCR4/CXCL12 and CXCR5/CXCL13 pathways. In particular, we observed a consistent reduction of the assessed receptors (CXCR4 and CXCR5) and a parallel increase of the corresponding ligands (CXCL12 and CXCL13) (Figure 5C,D,E,F). Conversely, no consistent modification in the expression of such markers was observed after DMARD treatment. Finally, neither RTX nor DMARD treatment led to a modulation of BAFF expression (Figure 5G).

## Discussion

To our knowledge, this is the first prospective, multi-center, follow-up study performed in a large cohort of active pSS patients, with recent disease onset for a period of 120 weeks, to assess safety and efficacy of RTX compared with DMARD treatment. Furthermore, we evaluated both histological and molecular patterns in the MSG tissue, induced by treatments.

One must point out that, in the last decades, therapeutic approaches in pSS have been based on the use of agents replacing the normal liquid secretion of the affected glands and glucocorticoids and immunosuppressive agents for extraglandular involvements. Available experimental data and systematic reviews highlighted the limited evidence for these drugs in pSS and the difficulties to suggest solid therapeutic recommendations [26]. Consequently, current therapeutic approaches are based on personal experience, expert opinion, and anecdotal studies, and until now no drugs have been identified to reduce disease activity or prevent damage and complications.

One limitation of this study is that the two treatment arms took place in different centers, thus potentially introducing bias. Despite the lack of a prospective randomization and two different sites of enrolment, we provided data showing that RTX treatment in patients affected by pSS is able to significantly decrease the ESSDAI when compared with the DMARD arm. Furthermore, this positive effect was observed throughout the entire study period, independently of the value of disease activity. As far as the safety of this treatment is concerned, RTX showed a good safety profile and no patients discontinued treatment or experienced side effects.

Depletion of B cells by RTX might reset immune tolerance and provide a new chance to an appropriate regulation of autoreactive B cells [48]. Moreover, depleting B cells might also influence the T-cell compartment, since B cells are effective antigen-presenting cells and can lead to autoantigen-specific >T-cell responses by providing co-stimulatory signals and secretion of cytokines and growth factors [49]. These data may partially explain the therapeutic efficacy of RTX treatment in autoimmune diseases [27].

Although few data are present in the literature to date, evidence from both open-label studies and randomized clinical trials provide the clue that RTX is effective in reducing different disease manifestations of pSS patients, such as sicca symptoms, extraglandular manifestations, fatigue and improving saliva production and eventually HR-QoL [28,30,31,36,38-40].

In our study, disease activity assessed by the ESSDAI appeared to be significantly reduced from baseline, starting from week 24 in both groups, but RTX was superior to DMARDs for improving the ESSDAI. This datum was partially due to a rapid and consistent score reduction of constitutional, lymphadenopathy, glandular, articular and cutaneous domains.

The second primary endpoint of this study was the safety of RTX treatment in these patients, and our study confirmed the safety profile of RTX therapy in pSS. No patients in our cohort showed systemic or infusion-related side effects and/or discontinued therapy. One must point out that we followed our patients for a very long-term period, performing six infusion courses in a period of 120 weeks. Furthermore, our patients did not develop any humoral immunodeficiency related to repeated treatment [50]. To our knowledge, no data are available in the literature concerning such a prolonged exposure to this drug in early pSS patients.

Concerning clinical assessment and secondary endpoints, we demonstrated a significant improvement of the VAS global disease activity, VAS pain, VAS fatigue, VAS dryness and VAS physician global assessment, when compared with baseline, and such improvement was significantly more pronounced in the RTX arm when compared with the DMARD arm. To note, the superiority of RTX was generally observed after two courses of therapy and continued throughout the study period. Concerning dryness, the RTX treatment group progressively improved from week 12 until the end of the study; on the contrary, in the DMARD treatment group we observed a slight improvement from week 12 that plateaued for the following period, and these data were confirmed by validated measures of salivary and lacrimal gland function. When we searched for a correlation between the extent of cytokine/chemokine modulation, disappearance of GCs and focus score, on one hand, and the clinical response, valuated by clinimetric tests, on the other, we did not find any significant correlation. Identification of potentially helpful biomarkers has been one of the main goals of the last two decades in autoimmunity, but the sample size of this study calculated to the assessed endpoints was unable to identify significant correlations.

Previous available studies in short series of pSS patients showed that beneficial effects occurred between 12 and 24 weeks after treatment and paralleled B-cell depletion in peripheral blood. Circulating CD19^+^ B cells started to repopulate at week 24, and 36 to 48 weeks after treatment were partially or fully reconstituted [32,40,51], describing only transient therapeutic effects, probably related to the relapse of the disease following B-cell repopulation. We speculate that the infusion regimen every 24 weeks prevented our patients developing disease flares secondary to the B-cell reconstitution. On these bases, RTX infusions (1,000 mg twice with an interval of 2 weeks) every 24 weeks in combination with long-term steroids might be a better strategy when compared with an on-demand regimen. Taken together, our data confirm that patients with early, active disease with extraglandular manifestations are likely to benefit from RTX treatment.

According to the aims of our study, we investigated whether, besides clinical efficacy, different systemic treatment may also change the extent of glandular involvement at baseline and week 120. We did not observe any statistically significant reduction in Chisholm and Mason score, focus score and GC presence in MSGs of patients treated with DMARDs. On the contrary, we observed that the presence of foci disappeared in 89.4% of RTX-treated patients after 120 weeks of treatment, thereby supporting the possibility to reverse from a FLS to a nonspecific chronic sialadenitis pattern or a full restoration of MSG architecture in these early patients. Furthermore we also observed that the number of GC-positive biopsies dropped from 52.6 to 5.2% in RTX-treated patients, and at the end of the study only one out of 19 patients still displayed GCs after RTX treatment. We might thus hypothesize that RTX is able to reduce glandular infiltrate and interfere with B-cell/T-cell compartmentalization, and consequently with the formation of ectopic lymphoid structures and GC-like structures in pSS MSGs.

In line with these data, we also observed a reduction of CXCR4-positive and CXCR5-positive cell percentage in total infiltrating mononuclear cells in RTX-treated but not in DMARD-treated patients at week 120 when compared with baseline. CXCR5 is expressed by naïve B cells, activated B cells, GC B cells, follicular B cells, follicular T cells, and CD4^+^ T cells, whereas CXCR4 is expressed on naïve B cells, memory B cells, GC B cells (mainly centroblasts), plasmablasts and plasma cells [52]. Their reduction might be partially due to the disappearance of B cells from MSGs and the reduction of inflammatory infiltrate. This reduction in the number of CXCR4-positive cells and CXCR5-positive cells was confirmed by the parallel reduction of CXCR4 and CXCR5 mRNA in MSGs of the RTX arm.

Furthermore, we found a statistically significant increase of CXCL12 and CXCL13 mRNA levels in the RTX arm, when compared with both baseline and the DMARD arm, at week 120. Thus, in this setting, we observed a consistent reduction of receptors (CXCR4 and CXCR5) and a parallel increase of corresponding ligands (CXCL12 and CXCL13). CXCL13, also known as B-lymphocyte chemoattractant-1, is produced by macrophages, T cells, and stromal, endothelial and ductal epithelial cells, and is present at very low levels in normal MSG biopsies [18,21]. CXCL13 is involved in the starting events leading to lymphoid organ formation and, mice lacking CXCL13 and its receptor CXCR5 show a partial blockade of lymph node organogenesis [53]. CXCL13 production is sustained by follicular dendritic cells, which play a pivotal role in the affinity maturation and antigenic selection of B cells within the GC. Furthermore, CXCL13 mainly drives GC B cells to the light zone where antigen selection occurs, and higher degree of lymphoid organization was found to be associated with an increased expression of this molecule [5]. CXCL12, or stromal cell-derived factor-1, is present in ductal epithelial cells and unlike CXCL13 was also found in MSG biopsies of healthy subjects. Concerning lymphoid structures, CXCL12 is present in the dark zone of GCs and, to note, was mainly associated with malignant CD20^+^ marginal zone-like B cells [54]. The chemokine–chemokine receptor pairs CXCL13–CXCR5 and CXCL12–CXCR4 might thus be closely associated with development of ectopic GC-like structures [5,21-23] as well as the peripheral B-cell abnormalities in pSS [25].

Increased levels of LTα and LTβ molecules, and their activation, have been associated with the presence of ectopic lymphoid structures at sites of chronic inflammation in several autoimmune diseases [20]. After treatment we observed that LTα and LTβ levels were significantly reduced at mRNA level in the RTX arm when compared with both baseline and the DMARD arm, and their amounts mirrored the values observed in healthy donor MSGs.

Taking together our molecular results, we might hypothesize that RTX treatment is able to deplete B cells in MSGs of pSS patients, but despite the observed reduction of inflammatory infiltrate, mainly B cells, and a disappearance of ectopic lymphoid structures, including a reduction of LTα and LTβ levels after RTX treatment, an abnormal micro-environment favoring B-cell homing in MSGs seems to persist.

This hypothesis is further supported by the observation that neither RTX or DMARD treatment led to a modulation of BAFF expression, which remains at the same level as at baseline. It is conceivable that, despite B-cell depletion, BAFF is not affected by any of these compounds. Several reports showed that BAFF in salivary glands appears to be produced not only by infiltrating B cells but also by T cells and ductal epithelial cells [55], primed by viruses, via the type 1 interferon pathway and/or viral Toll-like receptor ligands [56,57].

The formation of tertiary lymphoid tissue is the result of a multi-step dynamic process involving immune cells, epithelial cells, high endothelial venules, chemokines, cytokines, growth factors and related receptors. To our knowledge, RTX treatment seems to be able to interfere with this process, not only depleting B cells but also tuning the delicate equilibrium between cells, molecules and receptors, partially affecting the pro-B-cell inflammatory milieu. In our study we provided, for the first time, evidence regarding the modulation, after RTX treatment, of ectopic expression of lymphotoxins and homeostatic chemokines such as CXCL13 and CXCL12 and the interaction with their receptors, CXCR5 and CXCR4 respectively.

## Conclusion

This paper reports the first prospective, multi-center, follow-up study performed in a large cohort of active pSS patients, with recent disease onset carried out for a period of 120 weeks, to assess safety and efficacy of RTX compared with DMARD treatment, and correlating the clinical response to the immune-histological and molecular patterns before and after treatments. Our study shows that B-cell depleting therapy by RTX offers a promising and safe treatment for these patients, significantly ameliorating clinical features, when compared with other therapies, and restoring B-cell disturbance, by reducing immune infiltrate and lymphoid organization in target tissues. In fact, this therapy is able to interfere with the formation of tertiary lymphoid tissue, not only depleting B cells but also tuning the delicate equilibrium between cells, molecules and receptors, partially affecting the pro-B-cell inflammatory milieu that is typical of the inflamed glands. To our knowledge this study provided, for the first time, evidence regarding the modulation, after RTX treatment, of ectopic expression of lymphotoxins and homeostatic chemokines such as CXCL13 and CXCL12 and the interaction with their receptors, CXCR5 and CXCR4 respectively. Further studies are in progress to overcome some limitations of this study, such as the small number of patients studied in each group and the lack of randomization, on one hand, and additional biomolecular information from using gene array techniques on the other.

## Abbreviations

BAFF: B-cell activating factor; DMARD: Disease-modifying anti-rheumatic drug; ESSDAI: EULAR Sjögren’s syndrome disease activity index; FLS: Focal lymphocytic sialadenitis; GC: Germinal center; HR-QoL: Health-related quality of life; LT: Lymphotoxin; MSG: Minor salivary gland; pSS: Primary Sjögren’s syndrome; RTX: Rituximab; VAS: Visual analogic scales.

## Competing interest

The authors declare that they have no competing interests.

## Authors’ contributions

FCa, PC, AMar and RGi designed the study and wrote the paper. FCa, AMar, PDB and AMan performed histological and molecular experiments. PR, OB, IP, VL, SA, AA, FCi and GT enrolled and assessed patients and collected samples. FCa, PC, AMar, AA, RGe, GT and RGi analyzed data and critically reviewed the results. All authors read and approved the final manuscript.

## References

[B1] Tzioufas AG Vlachoyiannopoulos PG Sjogren’s syndrome: an update on clinical, basic and diagnostic therapeutic aspects J Autoimmun 2012 15 1 3 10.1016/j.jaut.2012.01.006 22361268

[B2] Garcia-Carrasco M Ramos-Casals M Rosas J Primary Sjogren syndrome: clinical and immunologic disease patterns in a cohort of 400 patients Medicine 2002 15 270 10.1097/00005792-200207000-00003 12169882

[B3] Fox RI Sjogren's syndrome Lancet 2005 15 321 331 10.1016/S0140-6736(05)66990-5 16039337

[B4] Meijer JM Meiners PM Slater JJRH Health-related quality of life, employment and disability in patients with Sjogren's syndrome Rheumatology 2009 15 1077 1082 10.1093/rheumatology/kep141 19553376

[B5] Barone F Bombardieri M Manzo A Blades MC Morgan PR Challacombe SJ Valesini G Pitzalis C Association of CXCL13 and CCL21 expression with the progressive organization of lymphoid-like structures in Sjogren’s syndrome Arthritis Rheum 2005 15 1773 1784 10.1002/art.21062 15934082

[B6] Bombardieri M Barone F Humby F Kelly S McGurk M Morgan P Challacombe S De Vita S Valesini G Spencer J Pitzalis C Activation induced cytidine deaminase expression in follicular dendritic cell networks and interfollicular large B cells supports functionality of ectopic lymphoid neogenesis in autoimmune sialoadenitis and MALT lymphoma in Sjogren’s syndrome J Immunol 2007 15 4929 4938 17878393 10.4049/jimmunol.179.7.4929

[B7] Anolik JH Looney RJ Lund FE Insights into the heterogeneity of human B cells: diverse functions, roles in autoimmunity, and use as therapeutic targets Immunol Res 2009 15 144 158 10.1007/s12026-009-8096-7 19350211 PMC2891332

[B8] Tatouli IP Tzioufas AG Pathogenetic aspects of humoral autoimmunity in Sjögren's syndrome Lupus 2012 15 1151 1154 10.1177/0961203312458717 22898537

[B9] Voulgarelis M Dafni UG Isenberg DA Moutsopoulos HM Malignant lymphoma in primary Sjögren’s syndrome: a multicenter, retrospective, clinical study by the European Concerted Action on Sjögren’s Syndrome Arthritis Rheum 1999 15 1765 1772 10.1002/1529-0131(199908)42:8<1765::AID-ANR28>3.0.CO;2-V 10446879

[B10] Theander E Henriksson G Ljungberg O Mandl T Manthorpe R Jacobsson LT Lymphoma and other malignancies in primary Sjogren's syndrome: a cohort study on cancer incidence and lymphoma predictors Ann Rheum Dis 2006 15 796 803 10.1136/ard.2005.041186 16284097 PMC1798187

[B11] Moisini I Davidson A BAFF: a local and systemic target in autoimmune diseases Clin Exp Immunol 2009 15 155 163 10.1111/j.1365-2249.2009.04007.x 19737141 PMC2768805

[B12] Thien M Phan TG Gardam S Excess BAFF rescues self-reactive B cells from peripheral deletion and allows them to enter forbidden follicular and marginal zone niches Immunity 2004 15 785 798 10.1016/j.immuni.2004.05.010 15189742

[B13] Groom J Kalled SL Cutler AH Association of BAFF/BLyS overexpression and altered B cell differentiation with Sjogren’s syndrome J Clin Invest 2002 15 59 68 11781351 10.1172/JCI14121 PMC150825

[B14] Lavie F Miceli-Richard C Quillard J Expression of BAFF (BLyS) in T cells infiltrating labial salivary glands from patients with Sjogren’s syndrome J Pathol 2004 15 496 502 10.1002/path.1533 15095277

[B15] Pers JO Daridon C Devauchelle V BAFF overexpression is associated with autoantibody production in autoimmune diseases Ann NY Acad Sci 2005 15 34 39 10.1196/annals.1313.004 16014518

[B16] Mariette X Roux S Zhang J The level of BLyS (BAFF) correlates with the titre of autoantibodies in human Sjogren’s syndrome Ann Rheum Dis 2003 15 168 171 10.1136/ard.62.2.168 12525388 PMC1754442

[B17] Szodoray P Alex P Jonsson MV Distinct profiles of Sjogren’s syndrome patients with ectopic salivary gland germinal centers revealed by serum cytokines and BAFF Clin Immunol 2005 15 168 176 10.1016/j.clim.2005.06.016 16126006

[B18] Corsiero E Bombardieri M Manzo A Bugatti S Uguccioni M Pitzalis C Role of lymphoid chemokines in the development of functional ectopic lymphoid structures in rheumatic autoimmune diseases Immunol Lett 2012 15 62 67 10.1016/j.imlet.2012.04.013 22698185

[B19] Aloisi F Pujol-Borrell R Lymphoid neogenesis in chronic inflammatory diseases Nat Rev Immunol 2006 15 205 217 10.1038/nri1786 16498451

[B20] van de Pavert SA Mebius RE New insights into the development of lymphoid tissues Nat Rev Immunol 2010 15 664 674 10.1038/nri2832 20706277

[B21] Amft N Curnow SJ Scheel-Toellner D Ectopic expression of the B cell–attracting chemokine BCA-1 (CXCL13) on endothelial cells and within lymphoid follicles contributes to the establishment of germinal center–like structures in Sjogren’s syndrome Arthritis Rheum 2001 15 2633 2641 10.1002/1529-0131(200111)44:11<2633::AID-ART443>3.0.CO;2-9 11710719

[B22] Xanthou G Polihronis M Tzioufas AG Paikos S Sideras P Moutsopoulos HM ‘Lymphoid’ chemokine messenger RNA expression by epithelial cells in the chronic inflammatory lesion of the salivary glands of Sjögren’s syndrome patients. Possible participation in lymphoid structure formation Arthritis Rheum 2001 15 408 418 10.1002/1529-0131(200102)44:2<408::AID-ANR60>3.0.CO;2-0 11229473

[B23] Salomonsson S Larsson P Tengnér P Mellquist E Hjelmström P Wahren-Herlenius M Expression of the B cell-attracting chemokine CXCL13 in the target organ and autoantibody production in ectopic lymphoid tissue in the chronic inflammatory disease Sjögren’s syndrome Scand J Immunol 2002 15 336 342 10.1046/j.1365-3083.2002.01058.x 11967114

[B24] Hansen A Reiter K Ziprian T Jacobi A Hoffmann A Gosemann M Scholze J Lipsky PE Dörner T Dysregulation of chemokine receptor expression and function by B cells of patients with primary Sjögren’s syndrome Arthritis Rheum 2005 15 2109 2119 10.1002/art.21129 15986367

[B25] Hansen A Lipsky PE Dörner T B cells in Sjögren’s syndrome: indications for disturbed selection and differentiation in ectopic lymphoid tissue Arthritis Res Ther 2007 15 218 10.1186/ar2210 17697366 PMC2206371

[B26] Ramos-Casals M Tzioufas AG Stone JH Siso´ A Bosch X Treatment of primary Sjögren syndrome, a systematic review JAMA 2010 15 452 460 10.1001/jama.2010.1014 20664046

[B27] Blüml S McKeever K B-cell targeted therapeutics in clinical development Arthritis Res Ther 2013 15 S4 10.1186/ar3906 23566679 PMC3624127

[B28] Meiners PM Vissink A Kallenberg CG Treatment of primary Sjogren’s syndrome with anti-CD20 therapy (rituximab): a feasible approach or just a starting point? Expert Opin Biol Ther 2011 15 1381 1394 10.1517/14712598.2011.605352 21819314

[B29] Kuijpers TW Bende RJ Baars PA CD20 deficiency in humans results in impaired T cell-independent antibody responses J Clin Invest 2010 15 214 222 10.1172/JCI40231 20038800 PMC2798692

[B30] St Clair EW Levesque MC Prak ET Vivino FB Rituximab therapy for primary Sjögren's syndrome: an open-label clinical trial and mechanistic analysis Arthritis Rheum 2013 15 1097 1106 10.1002/art.37850 23334994 PMC3618621

[B31] Gottenberg JE Cinquetti G Larroche C Combe B Hachulla E Meyer O Efficacy of rituximab in systemic manifestations of primary Sjogren's syndrome: results in 78 patients of the AutoImmune and Rituximab registry Ann Rheum Dis 2013 15 1026 1031 10.1136/annrheumdis-2012-202293 23264337

[B32] Pers JO Devauchelle V Daridon C BAFF-modulated repopulation of B lymphocytes in the blood and salivary glands of rituximab-treated patients with Sjogren’s syndrome Arthritis Rheum 2007 15 1464 1477 10.1002/art.22603 17469105

[B33] Pollard RPE Abdulahad WH Vissink A Hamza N Serum levels of BAFF, but not APRIL, are increased after rituximab treatment in patients with primary Sjögren’s syndrome: data from a placebo-controlled clinical trial Ann Rheum Dis 2013 15 146 148 10.1136/annrheumdis-2012-202071 22851468

[B34] Rosengren S Wei N Kalunian KC CXCL13: a novel biomarker of B-cell return following rituximab treatment and synovitis in patients with rheumatoid arthritis Rheumatology 2011 15 603 610 10.1093/rheumatology/keq337 21098574

[B35] Pijpe J van Imhoff GW Spijkervet FK Roodenburg JL Wolbink GJ Mansour K Rituximab treatment in patients with primary Sjogren’s syndrome: an open-label phase II study Arthritis Rheum 2005 15 2740e50 16142737 10.1002/art.21260

[B36] Devauchelle-Pensec V Pennec Y Morvan J Pers JO Daridon C Jousse-Joulin S **Improvement of Sjogren’s syndrome after two infusions of rituximab (anti-CD20)** Arthritis Rheum 2007 15 310e7 17330280 10.1002/art.22536

[B37] Jousse-Joulin S Devauchelle-Pensec V Morvan J Ultrasound assessment of salivary glands in patients with primary Sjogren’s syndrome treated with rituximab: quantitative and Doppler wave form analysis Biologics 2007 15 311 319 19707340 PMC2721315

[B38] Gottenberg JE Guillevin L Lambotte O Combe B Allanore Y Cantagrel A Tolerance and short term efficacy of rituximab in 43 patients with systemic autoimmune diseases Ann Rheum Dis 2005 15 913e20 15550531 10.1136/ard.2004.029694 PMC1755517

[B39] Galarza C Valencia D Tobon GJ Zurita L Mantilla RD Pineda-Tamayo R Should rituximab be considered as the first-choice treatment for severe autoimmune rheumatic diseases? Clin Rev Allergy Immunol 2008 15 124e8 18270866 10.1007/s12016-007-8028-z

[B40] Meijer JM Meiners PM Vissink A Effectiveness of rituximab treatment in primary Sjogren’s syndrome: a randomized, double-blind, placebo-controlled trial Arthritis Rheum 2010 15 960 968 20131246 10.1002/art.27314

[B41] Vitali C Bombardieri S Jonsson R Moutsopoulos HM Alexander EL Carsons SE Classification criteria for Sjogren’s syndrome: a revised version of the European criteria proposed by the American-European Consensus Group Ann Rheum Dis 2002 15 554 558 10.1136/ard.61.6.554 12006334 PMC1754137

[B42] Seror R Ravaud P Bowman SJ EULAR Sjögren’s Syndrome Disease Activity Index (ESSDAI): development of a consensus systemic disease activity index for primary Sjögren’s syndrome Ann Rheum Dis 2010 15 1103 1109 10.1136/ard.2009.110619 19561361 PMC2937022

[B43] Giusti L Baldini C Bazzichi L Ciregia F Tonazzini I Proteome analysis of whole saliva: a new tool for rheumatic diseases-the example of Sjögren's syndrome Proteomics 2007 15 1634 1643 10.1002/pmic.200600783 17436266

[B44] Hernández-Molina G Sánchez-Hernández T Clinimetric methods in Sjögren's syndrome Semin Arthritis Rheum 2013 15 627 639 10.1016/j.semarthrit.2012.09.008 23352255

[B45] Chisholm DM Mason DK Labial salivary gland biopsy in Sjogren’s disease J Clin Pathol 1968 15 656 660 10.1136/jcp.21.5.656 5697370 PMC473887

[B46] Daniels TE Whitcher JP Association of patterns of labial salivary gland inflammation with keratoconjunctivitissicca: analysis of 618 patients with suspected Sjogren’s syndrome Arthritis Rheum 1994 15 869 877 10.1002/art.1780370615 8003059

[B47] Ciccia F Giardina A Rizzo A Guggino G Cipriani P Carubbi F Giacomelli R Triolo G Rituximab modulates the expression of IL-22 in the salivary glands of patients with primary Sjogren's syndrome Ann Rheum Dis 2013 15 782 783 10.1136/annrheumdis-2012-202754 23264342

[B48] Leandra MJ B-cell subpopulations in humans and their differential susceptibility to depletion with anti-CD20 monoclonal antibodies Arthritis Res Ther 2013 15 S3 23566754 10.1186/ar3908 PMC3624669

[B49] Duddy ME Alter A Bar-Or A Distinct profiles of human B-cell effector cytokines: a role in immune regulation? J Immunol 2004 15 3422e7 15004141 10.4049/jimmunol.172.6.3422

[B50] Popa C Leandro MJ Cambridge G Edwards JC Repeated B lymphocyte depletion with rituximab in rheumatoid arthritis over 7 yrs Rheumatology (Oxford) 2007 15 626 630 17189244 10.1093/rheumatology/kel393

[B51] Abdulahad WH Meijer JM Kroese FG B-cell reconstitution and T-helper-cell balance after rituximab treatment of active primary Sjogren’s syndrome Arthritis Rheum 2011 15 1116 1123 10.1002/art.30236 21225693

[B52] Caron G LeGallou S Lamy T Tarte K Thierry Fest T CXCR4 expression functionally discriminates centroblasts versus centrocytes within human germinal center B cells J Immunol 2009 15 7595 7602 10.4049/jimmunol.0804272 19494283

[B53] Cupedo T Mebius RE Role of chemokines in the development of secondary and tertiary lymphoid tissues Semin Immunol 2003 15 243 248 10.1016/j.smim.2003.08.002 15001173

[B54] Barone F Bombardieri M Rosado MM Morgan PR Challacombe SJ De Vita S CXCL13, CCL21, and CXCL12 expression in salivary glands of patients with Sjogren’s syndrome and MALT lymphoma: association with reactive and malignant areas of lymphoid organization J Immunol 2008 15 5130 5140 18354239 10.4049/jimmunol.180.7.5130

[B55] Daridon C Devauchelle V Hutin P Aberrant expression of BAFF by B lymphocytes infiltrating the salivary glands of patients with primary Sjogren’s syndrome Arthritis Rheum 2007 15 1134 1144 10.1002/art.22458 17393395

[B56] Ittah M Miceli-Richard C Gottenberg EJ B cell-activating factor of the tumor necrosis factor family (BAFF) is expressed under stimulation by interferon in salivary gland epithelial cellsin primary Sjogren’s syndrome Arthritis Res Ther 2006 15 R51 10.1186/ar1912 16507175 PMC1526588

[B57] Ittah M Miceli-Richard C Gottenberg JE Viruses inducehigh expression of BAFF by salivarygland epithelial cells through TLR- and type-I IFN-dependent and -independent pathways Eur J Immunol 2008 15 1058 1064 10.1002/eji.200738013 18350548

